# Study on the Expression and Clinical Significances of Lewis y Antigen and Integrin αv, β3 in Epithelial Ovarian Tumors

**DOI:** 10.3390/ijms12063409

**Published:** 2011-05-27

**Authors:** Yifei Wang, Juanjuan Liu, Bei Lin, Changzhi Wang, Quanrong Li, Shuice Liu, Limei Yan, Shulan Zhang, Masao Iwamori

**Affiliations:** 1 Department of Obstetrics and Gynecology, Shengjing Hospital Affiliated to China Medical University, Shenyang 110004, China; E-Mails: converting@163.com (Y.W.); juanjuanliu_lg@yahoo.com.cn (J.L.); dwli_ll@163.com (Q.L.); liushuicc@sina.com (S.L.); limeimei@126.com (L.Y.); zhangshl99@126.com (S.Z.); 2 Department of Obstetrics and Gynecology, the Second Affiliated Hospital of Dalian Medical University, Dalian 116027, China; E-Mail: changzhiw@163.com; 3 Department of Biochemistry, Faculty of Science and Technology, Kinki University, 3-4-1 Kowakae, Higashiosaka, Osaka 577-8502, Japan; E-Mail: iwamori@163.com

**Keywords:** epithelial ovarian tumor, integrin αvβ3, Lewis y antigen, immunohistochemistry, immunofluorescence double labeling method

## Abstract

**Objective:**

To detect the expression and clinical significances of Lewis y antigen and integrin αv, β3 in epithelial ovarian tumors, and to explore the expression correlation between Lewis y antigen and integrin αv, β3.

**Methods:**

Immunohistochemical staining was performed in 95 cases of epithelial ovarian cancer, 37 cases of borderline tumors, 20 cases of benign tumors, and 20 cases of normal ovarian tissue, for the detection of Lewis y antigen and integrin αv, β3 expressions, and to analyze the relationship between Lewis y antigen and integrin, and the relationship between clinical and pathological parameters of ovarian cancer. In addition, immunofluorescence double labeling was utilized to detect the expression correlation between Lewis y antigen and integrin αv, β3 in ovarian cancer.

**Results:**

In epithelial ovarian tumors, the expression rate of Lewis y antigen was 81.05%, significantly higher than that of borderline (51.53%) (*P* < 0.05) and benign (25%) (*P* < 0.01) tumors, and normal ovarian tissues (0) (*P* < 0.01). The expression rate of integrin αv, β3 in malignant epithelial ovarian tumors was 78.95% and 82.11%, respectively, significantly higher than that of the borderline (45.94%, 40.54%) (both *P* < 0.05), benign group (10.00%, 15.00%) (both *P* < 0.01) and normal ovary group (5%, 15%) (both *P* < 0.01).

**Conclusions:**

Lewis y and integrins αv, β3 are relevant to pelvic and abdominal diffusion and metastasis of ovarian cancer cells, suggesting that these two molecules mediate a boosting function for tumor metastasis.

## 1. Introduction

Ovarian cancer is one of the most common malignancies among women, with an incidence that is on the rise, making it a serious danger to female health. In recent years, rapid progress has been made in glycobiology, particularly in the area of glycoconjugates (glycoproteins, glycolipids and proteoglycans) on the cell surface, which have been shown to be associated with many physiological functions and pathological mechanisms *in vivo*, such as growth and development, organization differentiation, protein intracellular transport, immune response, inflammation, carcinogenesis, *etc*. [1]. The relationships between glycoconjugates and tumors have received the most attention. Researchers have demonstrated that the malignant behaviors of tumor cells, such as invasion and metastasis, are all closely related to structure changes of sugar chains, which are the main component of glycoconjugates on the cell surface. Significant changes in the structure of the sugar chain on the membrane surface have been observed in the malignant process, affecting the structure and function of glycolipids and glycoproteins, both of which carry sugar chains [2].

Lewis y belongs to the Lewis blood group antigen family, which is a member of the blood group related antigens including A, B, H and Lewis antigen family. Lewis y antigen is located on the molecular ends of glycoproteins and glycolipids on the cell surface and belongs to oligosaccharides of two-fucose glycosylation. Lewis y antigen is expressed in early development of embryos, and its expression is limited to white blood cells and some epithelial cells in adults [3]. Lewis y antigen is expressed in some normal epithelial tissues and their secretions, however, overexpression is observed on the cell surface of most glandular epithelial origin tumors, including breast, ovarian, pancreatic, prostate, colorectal and non-small cell lung cancers [4]. Lewis y antigen expression is closely related to malignant processes and tumor cell proliferation, differentiation, invasion and metastasis behaviors.

As an important component of adhesion molecules, integrin molecules mediate cell–cell and cell–extracellular matrix (ECM) interactions, which play many important roles in tumor growth, invasion, metastasis, and drug resistance [5]. Our previous study confirmed that the structure and expression of Lewis y antigen are significantly correlated with integrin α5β1, and that Lewis y antigen plays an important role in ovarian cancer adhesion and resistance mediated by integrin α5β1 [6]. Our results show a close relationship between the Lewis y antigen and the integrin family. Another important member of the integrin family, integrin αvβ3, is highly expressed on the surface of many cancer cells [7].

In this study, we used immunohistochemistry to explore the expression pattern of Lewis y and integrins αvβ3, as well as their correlation in ovarian malignant epithelial tumors, borderline tumors, benign and normal ovarian tissues, and to analyze their relationship with clinicopathological parameters of ovarian cancer. Immunofluorescence double labeling was performed to further confirm the correlation between the Lewis y and integrin αv, β3. Our study provides a theoretical mechanism and a possible target for the development of future of biological treatment for ovarian cancer.

## 2. Results

### 2.1. Expression of Lewis y Antigen, Integrin αv and β3 in Different Ovarian Tissues

Lewis y antigen was expressed in the cytoplasm and cell membrane, with the majority of Lewis y on membrane, with a lack of staining in the nucleus. Lewis y antigen positive expression rate was 81.05% in the epithelial ovarian tumor group, significantly higher than that of the borderline group (51.35%) (*P* < 0.05) and the benign group (25.00%) (*P* < 0.01). Lewis y antigen positive rate was higher in the borderline group than in the benign group, with no statistically significant difference (*P* > 0.05). No Lewis y antigen expression was detected in the 20 normal ovary tissues, as shown in Table 1.

The expression of integrin αv and β3 were mainly on membrane, which is similar to Lewis y. The integrin αv positive expression rate was 78.95% in the ovarian malignant group, significantly higher than that of the borderline group (45.94%), the benign group (10.00%), and normal ovarian tissue (5.00%) (all *P* < 0.01). The positive expression rate of the borderline group was significantly higher than that of the benign group and normal ovarian tissues (all *P* < 0.01). There was no significant difference between benign and normal ovarian tissues (*P* > 0.05).

**Figure 1:**
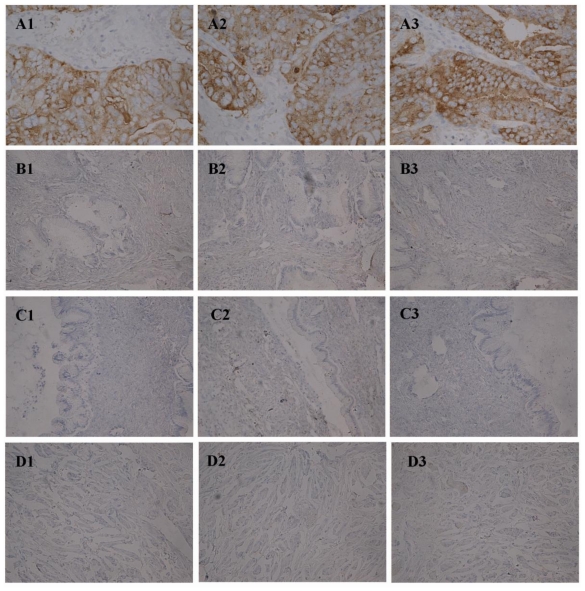
Immunohistochemical staining in ovarian malignant tumor (**A1**–**A3**), borderline tumor (**B1**–**B3**), benign tumor (**C1**–**C3**) and normal ovarian tissue (**D1**–**D3**). Integrin αv (**A1**, **B1**, **C1**, **D1**); β3 (**A2**, **B2**, **C2**, **D2**) and Lewis y (**A3**, **B3**, **C3**, **D3**). (Original magnification ×200).

The positive expression rate of integrin β3 in the ovarian cancer group (82.11%) was significantly higher than that of the borderline group (40.54%) (*P* < 0.05), the benign group (15.00%) (*P* < 0.01) and normal ovarian tissues (15.00%) (*P* < 0.01). The positive expression rate of the ovarian borderline group was significantly higher than that of the benign group and normal ovarian tissues (all *P* < 0.05). There was no significant difference in β3 positive expression rate between benign and normal ovarian tissue (Table 2, Figure 1).

### 2.2. Expression of Lewis y Antigen and Integrin αv, β3 and Their Relationships with Clinical and Pathological Parameters of Ovarian Cancer

In 95 patients with ovarian cancer, the Lewis y antigen positive rate in the abdominal metastasis group (91.67%) was higher than that in the group without abdominal metastasis (62.85%), with statistically significant difference (*P* < 0.01). The Lewis y positive expression rate of the ovarian cancer with high-moderate differentiated and poorly differentiated groups were 58.82%, 88.89%, respectively, with a statistically significant difference (*P* < 0.05). The Lewis y expression rate was significantly higher in the CA125-positive group (86.67%) than in the CA125 negative group (60.00%) (*P* < 0.05). Lewis y positive expression rate was higher in ovarian cancer III-IV stage (90.00%) than in the I-II stage (78.67%), but the difference between two groups was not statistically significant (*P* > 0.05) (Table 3).

Integrin αv expression rates in the abdominal metastasis group (96.67%) was significantly higher than that in the group without abdominal metastasis (48.57%) (*P* < 0.01). The integrin αv positive expression rate was 73.53% and 92.59% in high-moderate differentiated and poorly differentiated groups, respectively, with the statistically significant difference (*P* < 0.05). The expression rate in the CA125-positive group (80.00%) was slightly higher than in the CA125-negative group (75.00%), with no significant difference (*P* > 0.05). The integrin αv expression rate in ovarian cancer III~IV stage (75.00%) was slightly lower than in the I~II stage (80.00%), with no significant difference (*P* > 0.05) (Table 4).

Expression of integrin β3 was significantly different only in the abdominal diffusion transfer (*P* < 0.01), and showed no correlation with clinical staging, CA125 expression or histological grade (*P* > 0.05) (Table 5).

Thus, abdominal metastasis has the highest degree of consistency among the clinical pathology parameters which were correlated with Lewis y, integrin αv and β3 expression in ovarian cancer.

### 2.3. Correlation Analysis between Expression of Lewis y Antigen and Integrin αv, β3 in Ovarian Cancer

A similar trend was seen in the expression of Lewis y antigen, integrin αv, β3 in 95 patients with ovarian cancer, according to the results of immunohistochemistry. In order to investigate the correlation between these two antigens, we performed Spearman correlation analysis for each set of data, which demonstrated a high degree of positive correlation between Lewis y and integrin αv, β3 subunits (all *P* < 0.001) (correlation coefficients were 0.731, 0.605, respectively) (Tables 6 and 7). The Kappa test revealed consistency between Lewis y antigen and integrin αv or β3 subunit expression (*P* = 0.000), while Lewis y showed a slightly higher correlation with integrin αv subunit than with β3 subunit.

**Figure 2:**
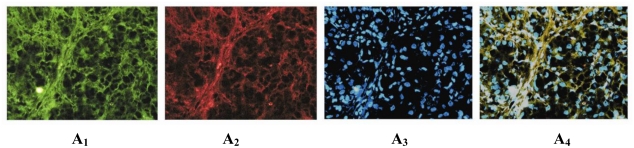

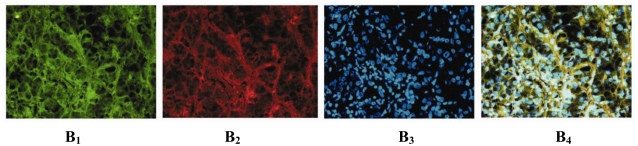
Integrin αv, β3 and Lewis y colocalize in ovarian malignant tumor. Using an immunofluorescent double-labeling method. Integrin αv and β3 (**A1** and **B1**); Lewis y (**A2** and **B2**); nucleus (**A3** and **B3**); Merged image (**A4** and **B4**). (Original magnification ×400).

In addition, immunofluorescence double-labeling revealed that in ovarian cancer Lewis y antigen (red fluorescence) was localized in the cell membrane and cytoplasm. Integrin αv and β3 (green fluorescence) were mainly localized in the cell membrane, with a small amount of coloring in the cytoplasm. The 4,6-diamino-2-phenyl indole (DAPI) (blue fluorescence) was used to visualize the nucleus. In three-channel synthesized images, the yellow fluorescence emerges from the area emitting both red and green fluorescence, indicating co-localization of Lewis y antigen and integrin αv, β3 (Figure 2).

## 3. Discussion

Glycoproteins, glycolipids and proteoglycans with differing compositions and structures exist on the cell surface, the sugar chains of which form antenna-like branches to receive information. These sugar chains play a key role as receptors for signaling molecules and are closely related to adhesion and recognition between cells, cell malignant transformation, invasion and metastasis. Lewis y is present in epithelial tissues, and during carcinogenisis, Lewis y expression was significantly increased in 60% to 90% patients with a poor prognosis [8], thus indicating it may serve as a marker for disease severity.

Yin *et al*. [9] found a positive expression rate and a strong positive expression rate of Lewis y antigen in ovarian carcinoma was 75% and 56%, respectively. Among 11 types of ovarian cancer cell lines tested, 7 cell lines were found to be Lewis y antigen positive. Baldus *et al*. [10] used immunohistochemistry to detect Lewis y antigen in 44 cases of colorectal adenocarcinoma and 42 cases of colorectal adenomas and found that the Lewis y antigen expression level was enhanced with adenoma degeneration and higher histological grade, suggesting that Lewis y antigen expression is related to the cell's degree of malignancy. Our previous work [11] also demonstrated that enhanced expression of Lewis y antigen increases the proliferation, adhesion, invasion, metastasis, drug resistance and other capacities of ovarian cancer cells. This study found the Lewis y antigen was highly expressed in epithelial ovarian cancer, with the positive rate of 81.05%, significantly higher than that of the borderline (51.35%) (*P* < 0.05), or the benign (25.00%) (*P* < 0.01), tumor group. In epithelial ovarian cancer, the Lewis y antigen positive rate in poorly differentiated group was 88.89%, significantly higher than in the high-moderate differentiated group (58.82%) (*P* < 0.05). It can be seen that the expression of the Lewis y antigen is increased with the increase of malignant degree, indicating a positive correlation between Lewis y antigen and ovarian cancer malignancy. Our results also showed that Lewis y expression is significantly related to the abdominal metastasis of epithelial ovarian cancer (*P* < 0.01). Taken together, these results indicate a correlation between the expression of Lewis y antigen and the occurrence and development of ovarian cancer.

The integrin family belongs to the cell adhesion molecules membrane receptor family, and can promote the growth, metastasis, angiogenesis and drug resistance of ovarian cancer. Due to its role in tumor cell adhesion, integrin overexpression may promote tumor cell invasion, adhesion and metastasis [12]. Integrin is also a mediator of angiogenesis, thereby promoting tumor growth, local invasion and distant metastasis. For instance, integrin αv, β3 is closely related to tumor invasion, metastasis and angiogenesis in ovarian cancer, malignant melanoma, breast cancer and prostate cancer [13]. Numerous studies have confirmed that integrin αv plays an important role in ovarian cancer progression and metastasis [14–17]. By activating integrin-linked kinase (IL-K), the integrin αv subunit promotes growth and proliferation of ovarian cancer cells, and mediates their adhesion and migration [18]. Integrin αv also promotes the adhesion of human ovarian epithelial cells (HOSE) to VN, is actively involved in cell proliferation and necessary for HOSE maximum proliferation [19]. Integrin αv, β3 overexpression significantly enhanced adhesion and proliferation of VN-dependent human ovarian cancer cell line OV-MZ-6, and enhanced the cell motility 5 folds in the adhesion of OV-MZ-6 cells to VN, accompanied by significant changes in cytoskeletal structure and cell morphology [20]. In this study, integrin αv, β3 expression was correlated to peritoneal metastasis, consistent with the Lewis y antigen results, suggesting that αv and β3 may both be involved in the mechanisms underlying peritoneal metastasis of ovarian cancer.

Researchers have shown that the mechanisms underlying changes in ovarian cancer physiological changes by Lewis y antigen are related to cell surface receptor proteins. Lewis y is connected to many end oligosaccharide chains of cell surface receptors, regulating cell biological behaviors through modification of cell surface receptors. In Lewis y antigen high expression tumor cells, Bsau *et al*. [21] have observed the presence of Lewis y antigen in epidermal growth factor receptor (EGFR). Klinger *et al*. [22] found that blocking Lewis y antigen with specific antibodies could affect the corresponding cell surface EGFR family-mediated cell signal transduction, changes in subcellular localization of signal substances, and accelerated cell surface recycling. Epithelial ovarian cancer tumor marker CA125 has also been shown to contain the Lewis y antigen structure [23]. In addition, the Lewis y expression rate in CA125-positive ovarian cancer was 86.67%, significantly higher than that in CA125-negative ovarian cancer (60.00%), with statistically significant difference (*P* < 0.05). The data from the current study indicate that Lewis y and CA125 are highly relevant, in agreement with the previous published results. Integrins are receptor proteins on the cell surface, with the extracellular matrix serving as their ligands. Our previous work demonstrated that the Lewis y structure existed in integrin α5β1 [6]. The results of this paper show that in epithelial ovarian cancer, Lewis y and integrin αv, β3 expression is highly relevant and consistent. Analysis showed a significant positive correlation between Lewis y antigen and integrin αv and β3 expression in ovarian cancer (*R* = 0.731, *P* = 0.000; *R* = 0.605, *P* = 0.000). Immunofluorescence double labeling indicates co-localization of Lewis y antigen and integrin αv, β3 in ovarian cancer tissues, further proving their close relationship.

Therefore, we can speculate that Lewis y antigens may be an important sugar chain structure associated with integrin αv, β3, and it can be exposed directly to the extracellular matrix, detecting extracellular microenvironment changes, thereby affecting integrin αv, β3 function and regulating ovarian cancer cell biological behaviors, including adhesion and migration.

## 4. Materials and Methods

### 4.1. Case Information and Tissue Samples

All clinical surgical tissues were collected from 2003 to 2009 in the Second Affiliated Hospital of Dalian Medical University and Dalian Maternity Hospital. All tissue slices were re-confirmed by pathology experts. There were 95 cases of primary epithelial ovarian cancer, 37 cases of borderline ovarian epithelial tumors, 20 cases of benign ovarian epithelial tumors, and 20 cases of normal ovarian tissue (from normal ovarian specimens cut in the same period of cervical cancer surgery), from patients ranging in age from 27 to 68 years (average 42.8 years). The clinical and pathological parameters of ovarian cancer patients include abdominal metastasis, clinical stage, histological grade, routine immunohistochemistry results of CA125. According to histological grade, among the 95 cases of ovarian cancer studied, there were 20 cases of high differentiated, 48 cases of moderate differentiated, and 27 cases of poorly differentiated. Clinical stage [according to International Federation of Gynecology and Obstetrics (FIGO) criteria]: 75 cases of I~II stage, 20 cases of III~IV stage. Among these 95 cases of ovarian cancer, 60 cases showed spread and metastasis in pelvic and abdominal cavities (including 20 cases uterine surface metastasis, 20 cases of tubal surface metastasis, and 20 cases of peritoneal surface metastasis). Seventy-five (75) cases were positive for CA125 expression.

### 4.2. Main Reagents

Rabbit anti-human integrin αv, β3 polyclonal antibody was purchased from Wuhan Boster Company, mouse anti-human Lewis y monoclonal antibody was purchased from Abcam Inc., biotinylated goat anti-mouse IgM, biotinylated goat anti-mouse IgG were purchased from Zhongshan Biotechnology Co., Ltd. Serum albumin (BSA) and DAB kit were purchased from Zhongshan Biotechnology Co., Ltd. Goat anti-rabbit IgG fluorescein isothiocyanate (FITC) and goat anti-mouse IgG tetraethyl rhodamine isothiocyanate (TRITC) were purchased from Fujian Wanxin company, and DAPI was purchased from Shenyang Baoxin company.

### 4.3. Immunohistochemistry

Streptavidin-biotin-peroxidase (SP) immunohistochemistry was performed. Tissues were fixed in 4% formaldehyde and embedded in paraffin, and 4 mm thick serial sections were prepared at the same organizational part. The working concentrations of Lewis y antibody and integrin αv, β3 antibody were 1:200 and 1:100, respectively. The staining procedure was performed according to SP kit manual. The group with PBS instead of primary antibody was used as a negative control. A colon cancer sample served as positive control for Lewis y antigen, and a breast cancer sample was a positive control for integrin αv, β3.

### 4.4. Immunofluorescence

The sample slices of strong expression for immunohistochemistry were selected to performed immunofluorescence double labeling method. Primary antibody combinations were anti-integrin αv with anti-Lewis y, or anti-integrin β3 with anti-Lewis y, with the PBS instead of primary antibody as the negative control. The working concentrations of rabbit anti-human integrin αv, β3 and mouse anti-human Lewis y antibody were all 1:100. The working concentrations of goat anti-rabbit IgM FITC and goat anti-mouse IgG TRITC were 1:100. The working concentration of nuclear dye DAPI was 1:100. The staining was performed according to the instructions of immunofluorescence kit.

### 4.5. The Determination of Results

The presence of brown colored granules on the cell membrane or in the cytoplasm was taken as a positive signal, and was divided by color intensity into not colored, light yellow, brown, tan and is recorded as 0, 1, 2, and 3, respectively. We choose five high-power fields in series from each slice, then score them and take the average percentage of chromatosis cells. A positive cell rate of less than 5% was a score of 0, a positive cell rate of 5~25% was a score of 1, a positive cell rate of 26~50% was a score of 2, positive cell rate of 51~75% was a score of 3, positive cell rate of more than 75% was a score of 4. The final score was determined by multiplying positive cell rate and score values: 0~2 was equal to negative expression (−), 3~4 was equal to weakly positive (+), 5~8 was equal to moderate positive (++), 9~12 was equal to strong positive (+++). The results were read by two independent observers to control for variability.

Microscopic red fluorescence indicated Lewis y antigen labeled by TRITC, green fluorescence indicated integrin αv, β3 labeled by FITC, while blue fluorescence indicated DAPI-stained nucleus. Pictures of the three individual fluorescence channels were superimposed using image analysis software, with a yellow fluorescence indicated co-localization of Lewis y antigen and integrin αv, β3.

### 4.6. Statistical Analysis

Statistical analyses were performed using the SPSS software Version 11.5. Data expressed as mean ± SD was applied for statistical analysis. The Student’s t test was applied to compare data between the two groups, and analysis of variance was applied to compare data among multiple groups. The Chi-square (χ^2^) test was applied to analyze the expression of Lewis y antigen, integrin αv, β3 and clinicopathological parameters. The Spearman correlation analysis method was applied to calculate the coefficient *R* of indexes and to analyze its correlation, A *P* value <0.05 was considered statistically significant.

## 5. Conclusions

In summary, Lewis y and integrins αv, β3 are relevant to pelvic and abdominal diffusion and metastasis of ovarian cancer cells, suggesting that these two molecules mediate a boosting function for tumor metastasis.

## Figures and Tables

**Table 1:** Expression of Lewis y antigen in different ovarian tissues.

Groups	Lewis y Antigen			
	N (cases)	−	+ ~ +++	Positive Rate (%)
malignant group	95	18	77	81.05 *
borderline group	37	18	19	51.35 **
benign group	20	15	5	25.00
normal group	20	20	0	0

* Note: malignant group is compared with other groups;

** borderline group is compared with benign group.

**Table 2:** Expression of integrin αv, β3 in different ovarian tissues.

Groups	Cases	Integrin αv				Positive Cases	Positive Rate (%)	Integrin β3				Positive Cases	Positive Rate (%)
		−	+	++	+++			−	+	++	+++		
malignant group	95	20	17	21	37	75	78.95 *	17	18	35	25	78	82.11 *
borderline group	37	20	7	7	3	17	45.94 *	22	6	5	4	15	40.54 *
benign group	20	18	2	0	0	2	10.0	17	2	1	0	3	15.00
normal group	20	19	1	0	0	1	5.00	17	2	1	0	3	15.00

* Note: malignant group, borderline group are compared with benign group and normal group, respectively.

**Table 3:** Relationship between Lewis y antigen with the clinical and pathological parameters of malignant ovarian cancer.

Characteristics	*n*	Lewis y			*P*	*X*^2^
		−	+ ~ +++	Positive Rate (%)		
**FIGO Stages**						
I~II	75	18	59	78.67	>0.05	1.736
III~IV	20	2	18	90.00		

**Histological Grades**						
high-moderate	68	28	40	58.82	<0.05	9.196
poorly	27	3	24	88.89		

**Abdominal Diffusion Metastasis**						
No	35	13	22	62.85	<0.01	11.947
Yes	60	5	55	91.67		

**CA125**						
negative	20	8	12	60.00	<0.05	7.311
positive	75	10	65	86.67		

**Table 4:** Relationship between integrin αv with the clinical and pathological parameters of malignant ovarian cancer.

Characteristics	*n*	Lewis y			*P*	*X*^2^
		−	+ ~ +++	Positive Rate (%)		
**FIGO Stages**						
I~II	75	15	60	80.00	>0.05	0.238
III~IV	20	5	15	75.00		

**Histological Grades**						
high-moderate	68	18	50	73.53	<0.05	4.226
poorly	27	2	25	92.59		

**Abdominal Diffusion Metastasis**						
No	35	18	17	48.57	<0.05	30.765
Yes	60	2	58	96.67		

**CA125**						
negative	20	5	15	75.00	>0.05	0.238
positive	75	15	60	80.00		

**Table 5:** Relationship between integrin β3 with the clinical and pathological parameters of malignant ovarian cancer.

Characteristics	*n*	Lewis y			*P*	*X*^2^
		−	+ ~ +++	Positive Rate (%)		
**FIGO Stages**						
I~II	75	13	62	82.67	>0.05	0.076
III~IV	20	4	16	80.00		

**Histological Grades**						
high-moderate	68	15	53	77.94	>0.05	2.824
poorly	27	2	25	92.59		

**Abdominal Diffusion Metastasis**						
No	35	15	20	51.74	<0.01	23.503
Yes	60	2	58	96.67		

**CA125**						
negative	20	5	15	75.00	>0.05	0.870
positive	75	12	63	84.00		

**Table 6:** Correlation between expression of Lewis y antigen and integrin αv in ovarian cancer.

Lewis y (*n*)	Integrin αv (*n*)				*R*	*P*
	− (20)	+ (17)	++ (21)	+++ (37)		
− (18)	12	3	2	1	0.731	0.000
+ (15)	5	8	1	1		
++ (20)	2	4	10	4		
+++ (42)	1	2	8	31		

**Table 7:** Correlation between expression of Lewis y antigen and integrin β3 in ovarian cancer.

Lewis y (*n*)	Integrin β3 (*n*)				*R*	*P*
	− (17)	+ (21)	++ (18)	+++ (39)		
− (18)	10	3	2	3	0.605	0.000
+ (15)	4	8	1	2		
++ (20)	2	4	11	3		
+++ (42)	1	6	4	31		
